# Nutrition policy or price stabilisation policy: which policy is more effective for nutrition outcomes?

**DOI:** 10.1186/s40795-024-00882-6

**Published:** 2024-05-11

**Authors:** Marilys Victoire Razakamanana, Miora Rakotonirainy, Tiarinisaina Olivier Ramiandrisoa

**Affiliations:** Centre de Recherche pour le Développement (CRD), Université Catholique de Madagascar Ambatoroka, Antananarivo 101, Ambatoroka, BP 6059 Madagascar

**Keywords:** Madagascar, Malnutrition, MCHW, Price stabilisation policy, Rice

## Abstract

**Background:**

Malnutrition remains a global problem and is increasing with the emergence of the COVID-19 pandemic. In Madagascar, half of the children under five years of age suffer from stunting. However, since 2006, vitamin A supplementation campaigns, deworming and free vaccinations have been implemented within the framework of the Mother and Child Health Week (MCHW) to strengthen the fight against micronutrient deficiencies and reduce mortality. On the other hand, rice, the staple food of the Malagasy population, can provide some of the micronutrients necessary for good nutrition. However, the country’s rice production is still insufficient, and the price has been rising steadily in recent years. This has led the government to resort to the policy of stabilizing rice prices through imported rice in 2017 and 2018. The aim of this paper is therefore to analyse the effects of these policies on the prevalence of malnutrition among children under five years of age in Madagascar. Which policy would be more effective: the nutrition policy or the price stabilisation policy?

**Methods:**

Data from the Multiple Indicator Cluster Survey conducted by the National Institute of Statistics in 2018 are used, and logistic regressions for the analysis of the effects of nutrition policies on nutrition outcomes are performed. For the effect of price stabilisation policy, panel data on 22 regions of Madagascar from 2016 to 2019 are considered, and a fixed effect model is used.

**Results:**

We found that the effects of the nutrition policy are not immediately visible. Only participation in the 2016 MCHW contributes to a lower probability of malnutrition occurrence. The odds ratios of the effects of this participation on stunting are 0.69 (p-value: 0.05); on underweight: 0.70 (p-value < 0.01); and on wasting: 0.57 (p-value < 0.01). However, the participation rate remains very low. Then, there is no effect of the rice price stabilization policy on nutrition outcomes (0.00; p-value = 0.11).

**Conclusions:**

Price stabilisation policy is not sufficient to fight against malnutrition, due to a lack of food diversification. These results suggest the need for a policy to ensure nutritional intake and to sensitize the population to participate in the MCHW.

**Trial registration:**

Not applicable.

**Supplementary Information:**

The online version contains supplementary material available at 10.1186/s40795-024-00882-6.

## Background

Good nutrition enables a healthy and productive life [1]. However, malnutrition increases the risk of mortality, stunted growth, and cognitive deficits in young children, leading to poor school performance and compromising future productivity in labour market [2]. Malnutrition includes undernutrition, overweight, obesity, and micronutrient deficiencies. It continues to affect millions of women and children, particularly in low- and middle-income countries [3]. According to the United Nations, SDG 2 on zero hunger is still far from being achieved. This is further compromised by the emergence of COVID-19. Before the pandemic, approximately 690 million people, or 8.9% of the world population, were undernourished [1]. According to the Food and Agriculture Organization (FAO) (2020) estimate, COVID-19 could lead to an additional 83–132 million undernourished people compared to 2019. According to FAO et al. (2021), 45% of deaths in children under 5 years of age are caused by undernutrition. These deaths occur particularly in low- and middle-income countries [4].

In Eastern and Southern Africa the number of stunted children has risen from 23.6 million to 26.8 million in 25 years. An estimated 1.8–2 million children aged 6–59 months require treatment for severe acute malnutrition in this region (UNICEF, 2023)^1^.

Madagascar is among the four (4) most hunger-affected countries in the world, with Chad, Yemen and Zambia [5]. The population suffers from chronic malnutrition, and the country is ranked third in sub-Saharan Africa in this area. In fact, 42% of children under the age of five years were affected, while 6% of children suffered from acute malnutrition in 2018 [6]. Note that chronic malnutrition involves stunting related to a food quality problem, while acute malnutrition or wasting results from recent and significant weight loss due to severe food starvation. More than half of the island’s regions have a prevalence of malnutrition higher than 40%, and the most affected regions are those of the highlands, where the rate is close to 60%.

With the adoption of the National Nutrition Policy (NNP) in 2004, the government has integrated the fight against malnutrition into the strategies of fighting against poverty. Within the framework of the NNP, concrete actions have been carried out through the National Action Plan for Nutrition. The objective is to reduce the chronic malnutrition rate among children under the age of five years from 47% in 2004 to 38% in 2021. In Madagascar, for example, the Mother and Child Health Week (MCHW), has been held twice a year throughout the country, since 2006. The MCHW aims to provide free vitamin A supplementation, deworming and vaccinations to strengthen the fight against micronutrient deficiency and to reduce maternal and child mortality. Regarding the rice sector, urban supply has been regulated by imports since 2000 [7]. The purpose of imports is first to compensate for the gap between production and local demand for rice, which is growing with the population but also to regulate the price of local rice. Between 2014 and 2019, Madagascar imported an average of 380,000 tons of rice per year. The peak occurred in 2017 with 570,000 tons of imported rice [8]. Despite these measures, the malnutrition rate remained high in 2018 (42%), and the situation did not improve until 2020 [6]. In addition, regional disparity has increased. The rate varies from 20 to 60% depending on the region. The regions of the central highlands are the most affected, and the southeast region is the least affected.

In view of this context, the general objective of this study is to analyse the impacts of national nutrition policies through the implementation of vitamin A supplementation, deworming and immunization campaigns during the MCHW in Madagascar. Then, we study the reasons for the regional disparity in terms of malnutrition in the country and analyse the effects of the price stabilisation policy via imports on nutrition outcomes. Rice is considered as it is the staple food of the Malagasy people. In addition, rice is an important source of carbohydrates and proteins as well as other essential nutrients, especially in developing countries [9, 10]. The purpose is to identify which policy is more effective in addressing malnutrition, direct nutrition policy or agricultural policy.

## Literature review

### Nutritional policy in Madagascar

The review of nutrition policies and programs implemented since independence highlights Madagascar’s institutional commitment to the fight against malnutrition, which has evolved over time and is increasingly linked to national development policy.

The establishment of the nutrition policy follows Madagascar’s participation in various international conferences on nutrition and its adherence to the Millennium Development Goals (MDGs) and the Sustainable Development Goals (SDGs). Therefore, the first National Nutrition Policy (NNP) was implemented in 2004. Its general objectives were to reduce by half the prevalence of chronic malnutrition among children under the age of five years.

Several programs and projects have been established to implement nutrition policy. From 1985 to 1989, there were projects on strengthening legislation and activities on nutrition (1985 to 1989) and on strengthening food control structures (1989 to 1992). In 1989, the country developed the Food and Nutritional Surveillance Program, followed by the National Program to fight against Nutritional Deficiencies (1990–1994).

Since 2006, Mother and Child Health Week (MCHW) has been implemented to ensure vitamin A supplementation and deworming of children under the age of five years and pregnant women. This campaign is organized twice a year across the country through the provision of a package of free interventions. In addition, routine immunization reinforcement sessions have been organized to improve the immunization coverage of children and pregnant women^2^.

### Rice production situation in Madagascar

The link between rice production and nutrition is not yet clear. Among the 22 regions of Madagascar, the central highlands composed of Vakinankaratra, Itasy, Alaotra Mangoro, Amoron’i Mania and Analamanga are the most affected by malnutrition. Nevertheless, these regions are the rice granaries of Madagascar, with an average production of 500,000 tons per year. In 2018, the region’s production was 540,000 tons (World Food Programme data, 2019).

As a staple food, most Malagasy households consume rice three times a day. The average annual consumption of rice per person is 130 kg [11], and the amount of rice produced remains below the amount consumed. This situation is explained by several factors, such as the low exploitation of arable land (only 10% of the 36 million hectares of arable land are cultivated), the insufficient level of development of hydroagricultural infrastructure, the use of rudimentary methods and the low level of development of road infrastructure, limiting foodstuff accessibility. To avoid malnutrition and to stabilize the price of rice following the insufficiency of supply compared to demand, the Malagasy government had to import rice. The main suppliers are India, Pakistan and Thailand [12]. Note that the price of imported rice is lower than the price of local rice and is 1,900 MGA, compared to approximately 2,300 MGA in December 2020 (data of the Ministry of Agriculture, Livestock and Fisheries, 2021). Thus, this policy of price stabilisation should also contribute to the fight against malnutrition in Madagascar.

### Impacts of nutrition and agricultural policies on nutrition outcomes

According to Luo et al. (2020), “Malnutrition in all its forms” can be measured using an index based on six global targets approved by the World Health Assembly that can show nutrition outcomes [13]. Indicators include stunting, wasting, overweight in preschool children, anaemia in women of childbearing age, low birth weight, and exclusive breastfeeding. Among micronutrient deficiencies, vitamin A deficiency is a major concern for children, especially those living in developing countries. According to UNICEF, approximately one-third of children do not receive the needed dose of vitamin A [14]. Children who did not receive foods rich in vitamin A were then more likely to be anemic [15].

Kangas et al. (2020) studied the impact of a therapeutic food program on children with acute malnutrition [16]. The treatment consisted of providing sufficient micronutrients and aimed to restore body reserves. However, the treatment has shown little success. In other words, vitamin A status did not differ between before and after treatment [16]. Thus, the authors suggested revising the treatment nutrients. However, Bhutta et al. (2013) showed that vitamin A supplementation reduced all-cause mortality by 24% and diarrhea-related mortality by 28% [17]. To strengthen this point, Keats et al. (2021) report that multiple micronutrient supplementation is highly effective for health [3].

Parasitic infections also cause anemia and limit the physical and cognitive development of children [18]. Sudarsanam and Tharyan (2013) studied the impact of deworming on infected children. The results showed that deworming increased children’s weight and eventually hemoglobin [18].

Then, Ecker & Qaim (2011) studied the impacts of income and price policies on nutrient consumption and the prevalence of nutritional deficiencies. The authors found that income-related policies are better suited than price-related policies to improve nutrition [19]. Pandey et al. (2016) analysed the impact of agricultural interventions on nutritional status in South Asia. The results showed that the production of targeted nutrient-rich crops and the diversification of agricultural production can potentially improve nutrient intake and nutrition outcomes [20].

Regarding the relationship between food commodity prices and nutrition, many studies that are generally based on household surveys have shown strong links between these variables. Recent studies on the rice market and policies in Indonesia have suggested that some level of liberalization, rather than stimulating production by raising domestic prices, could help the government achieve nutrition objectives [21, 22]. Ecker & Qaim (2011) argued that a price shock in the main staple food would have a considerable impact on diets, with particularly strong effects on food buyers in rural areas [19]. Anríquez et al. (2013), in their study of eight developing countries, found that simulated food price spikes reduce average consumption and food intake and worsen the distribution of food calories [23].

### Other determinants of malnutrition

Regarding the other determinants of malnutrition, the characteristics of the child can explain malnutrition, such as his health. Ake Tano et al. (2010) associated fever and diarrhea with chronic malnutrition. Indeed, diseases cause a loss of nutrient intake, as well as a disorder of metabolism [24].

Malnutrition can also depend on the gender of the child. In their studies, Kobelembi (2004), Mukalay et al. (2010), Black et al. (2013) and Augsburg & Rodriguez-Lesmes (2018) found that male children are more susceptible to malnutrition [17, 25–27]. In addition, many studies have shown that the risk of stunting increases with age [26].

Regarding the mother’s variables, her body mass index (BMI) [17, 24], education level [28] and age [25] are associated with child stunting. Breastfeeding is one of the determining factors of a child’s nutritional status. It provides the child with all the nutritional needs necessary for growth until the age of 6 months. However, its importance goes beyond this age [24, 29].

Other household characteristics, such as sanitation, use of “safe” toilets [29], access to safe water [26] and access to health care [30], can also explain chronic malnutrition in children. Finally, disparities in activity and lifestyle in urban and rural areas translate into unequal risks to exposure to malnutrition. Urban areas are characterized by the presence of health infrastructure and a larger number of health professionals than rural areas. For Waihenya et al. (1996) and Fagbamigbe et al. (2020), rural areas are more susceptible to malnutrition than urban areas [31, 32].

To summarize, Harris & Nisbett (2020) described the fundamental determinants of nutrition. The authors identified three interrelated factors. First, the resources that demonstrate the potential of individuals and groups to benefit from goods and services such as purchasing food and accessing appropriate health care. Second, the structures of society that condition access to these nutrition-related goods and services. Third, social patterns (gender, ethnicity, religion, age, disability) produce inequalities for some groups [33].

## Methodology

### Analysis of the effects of nutrition policies on nutrition outcomes

#### Data

We use data from the Multiple Indicator Cluster Survey (MICS) in Madagascar in 2018. This is a household survey conducted by INSTAT and supported by UNICEF. A two-stage stratified sampling method was used to select the survey sample. The total sample size for the MICS Madagascar 2018 survey is 20,000 households, comprising a minimum sample of 800 households per region and a maximum of 1,200 households for the largest regions [8]. For children under the age of five years, the data consist of 14,431 observations and include information on children aged 0 to 4 years. To estimate the sample size for this survey, we have:

$$n=\frac{\left[4\left(r\right)\left(1-r\right)\left(deff\right)\right]}{\left[{\left(RME*r\right)}^{2}\left(pb\right)\left(AveSize\right)\left(RR\right)\right]}$$

where:

n: needed sample size, expressed in number of households.

4: factor to reach the 95% confidence level.

r: predicted or expected value of the indicator, expressed as a proportion.

deff: design effect for the indicator.

RME: relative margin of error to be tolerated at the 95% confidence level, defined as a percentage of r for national level estimates.

pb: proportion of the total population on which the indicator r is based.

AveSize: average household size (number of persons per household).

RR: expected response rate.

According to the 2008–2009 DHS, the indicator (r) was 43.9%; the proportion of women who gave a live birth in the five years preceding the survey to the total population (pb) was 10.6%; and the average household size (AveSize) was 4.7. The design effect (deff) is set at 2; the relative margin of error to be tolerated at the 95% confidence level is 0.16, or an absolute margin of error of 0.16 × 0.439 = 0.07; the expected response rate (RR) is 90%. To have representativeness at the 22-region level, the size n is multiplied by 22 [8].

#### Dependent variables

The three anthropometric indicators most commonly used to measure malnutrition in children are stunting (low height for age) (HAZ), underweight (low weight for age) (WAZ) and wasting (low weight for height) (WHZ) [34].

The MICS provides these indicators. Weight-for-age (WAZ) is a measure of both acute and chronic malnutrition. Children whose WAZ ratio is more than two standard deviations below the median of the reference population are considered moderately or severely underweight, while those whose weight-for-age ratio is more than three standard deviations below the median are considered underweight.

The height-for-age (HAZ) is a measure of linear growth. Children whose height-for-age is more than two standard deviations below the median of the reference population are considered short for their age and may be classified as moderately or severely stunted. Those whose height-for-age ratio is more than three standard deviations below the median are classified as severely stunted.

The weight-for-height (WHZ) is used to assess wasting and overweight status. Children whose weight-for-height ratio is more than two standard deviations below the median of the reference population are classified as moderately or severely emaciated, while those more than three standard deviations below the median are considered severely emaciated.

We consider binomial malnutrition variables (Model 1). The critical points of the z-score are used to classify children according to their nutritional status. Thus, children suffering from stunting or wasting or underweight are malnourished, and we take the value 1 for malnutrition and 0 otherwise.

Then, for the indicators related to stunting, underweight and wasting, we consider an ordinal scale based on z-scores (Models 2, 3 and 4). The modalities for stunting, underweight and wasting are 0: representing good health (z-score > 0), 1: representing moderate malnutrition (-2 < z-score < 0), 2 - representing malnutrition (-3 < z-score < -2) and 3: representing severe malnutrition (z-score < -3). The reference value for comparison will be 0 (good health).

#### Models

This paper use the model of Feyisa et al. (2023) to identify the determinants of malnutrition [35].

As variables of interest, participation in MCHW in 2016, 2017, and 2018 (yes: 1; no: 0).

As control variables:

- Child characteristics: age, sex (male: 0; female: 1).
- Mother’s characteristics: level of education (0: illiterate or preschool; 1: primary; 2: secondary and above), base being 0 illiterate, marital status (in couple: 1; alone: 0).
- Household characteristics:
- residence (1: urban, 2: rural), the base is urban;
- region, out of the 22 regions noted from 1 to 22, we considered as base the Analamanga region insofar as the capital of Madagascar and where the policies are decided (region 1);
- income quintile (1: poorest, 2: poor, 3: average, 4: rich, 5: richest), the reference is the poorest, the majority of Malagasy (75%) are in this category (World Bank, 2020);
- source of drinking water (1: improved, 0: unimproved),
- type of toilet (1: improved, 0: unimproved).
- type of cooking fuel (1: modern, 0: traditional).

Improved water and toilet sources follow the WHO (2023) definition. Traditional cooking fuel types include those using biomass, while others using gas, electricity and oil are considered modern [36].

Logistic regressions are performed. For model 1, measuring the presence of malnutrition or not, a binary logistic regression is used.

$$\eqalign{ Logit\,\left[ {P({Y_i} = 1\left| {X = {x_1} \ldots {x_n})} \right.} \right]\, & \cr & = \,{a_0} + {a_1}{x_1} + ... + {a_n}{x_n} \cr}$$

With Y: the presence or absence of malnutrition.

X: the independent variables.

For models 2 and 3, ordinal logistic regressions are performed.

$${ln}\left[\frac{P(Y=\_1/X}{P(Y=\_0/X}\right]={a}_{0}+{a}_{1}{x}_{1}+?+{a}_{n}{x}_{n}$$

$${ln}\left[\frac{P(Y=\_2/X}{P(Y=\_0/X}\right]={a}_{0}+{a}_{1}{x}_{1}+\dots +{a}_{n}{x}_{n}$$

$${ln}\left[\frac{P(Y=\_3/X}{P(Y=\_0/X}\right]={a}_{0}+{a}_{1}{x}_{1}+\dots +{a}_{n}{x}_{n}$$

Y: ordinal scales for stunting (Model 2), underweight (Model 3), and wasting (Model 4).

X: Independent variables.

Finally, for the robustness test, the probit method is used (Appendix 1).

### Analysis of the effects of price stabilisation policy on malnutrition

Insofar as the use of rice imports constitutes a policy to stabilize the price of this product in Madagascar, we consider the evolution of the price of rice and production by region as variables of interest during the period 2016–2019. Our basic model (Model 1) is as follows:

$$\eqalign{{M_{it\,}} = \,{{\rm{\beta }}_{\rm{1}}}\, + \,{{\rm{\beta }}_{\rm{2}}}{P_{it}}\, + \,{{\rm{\beta }}_{\rm{3}}}{Q_{it}}\,\ + \,{{\rm{\beta }}_{\rm{4}}}X{}_{it}\, + \,{{\rm{\beta }}_{\rm{5}}}{Z_t}\, + \,{\varepsilon _{it}} \cr}$$

- M is the malnutrition rate;
- P, the price of rice;
- Q, local rice production.
- X are variables that can explain malnutrition, such as the Human Development Index by region according to Ahsan & Maharaj (2018) [28] and the proportion of urban and rural population by region [37].
- t is the time, i.e., the years 2016 to 2019.
- i are the 22 regions of Madagascar.

M and X come from Global Lab Data (2021), and price and production of rice by region from the Ministry of Economy and Finance of Madagascar. We do not consider the 2020 period because the occurrence of the pandemic could bias our results.

Since we have panel data composed of 88 observations (4 years*22 regions), depending on the result of the Hausman test, the method to be used will be a fixed or random effects model. Indeed, the choice of model in panel data must be based on information about the individual specific components and the exogeneity of the independent variables. The Hausman test is used for testing whether fixed or random effects model is appropriate, by identifying the presence of endogeneity in the explanatory variables. In the null hypothesis of the Hausman, the appropriate model is random effect, there is no correlation between the error term and the independent variables in the panel data model. In the alternative hypothesis, the appropriate model is fixed effect. The correlation between the error term and the independent variables in the panel data model is statistically significant [38].

To check the robustness of the relationship, instead of considering production and price separately, we considered the variable production multiplied by price (P*Q) (Model 2).

## Results

### Impact of MCHW on the nutritional status of children under five

#### Descriptive analyses

MICS data show that 50.70% of children are male and 49.30% are female. The average age of the children was 2 years (SD: 1.4).

**Fig. 1:**
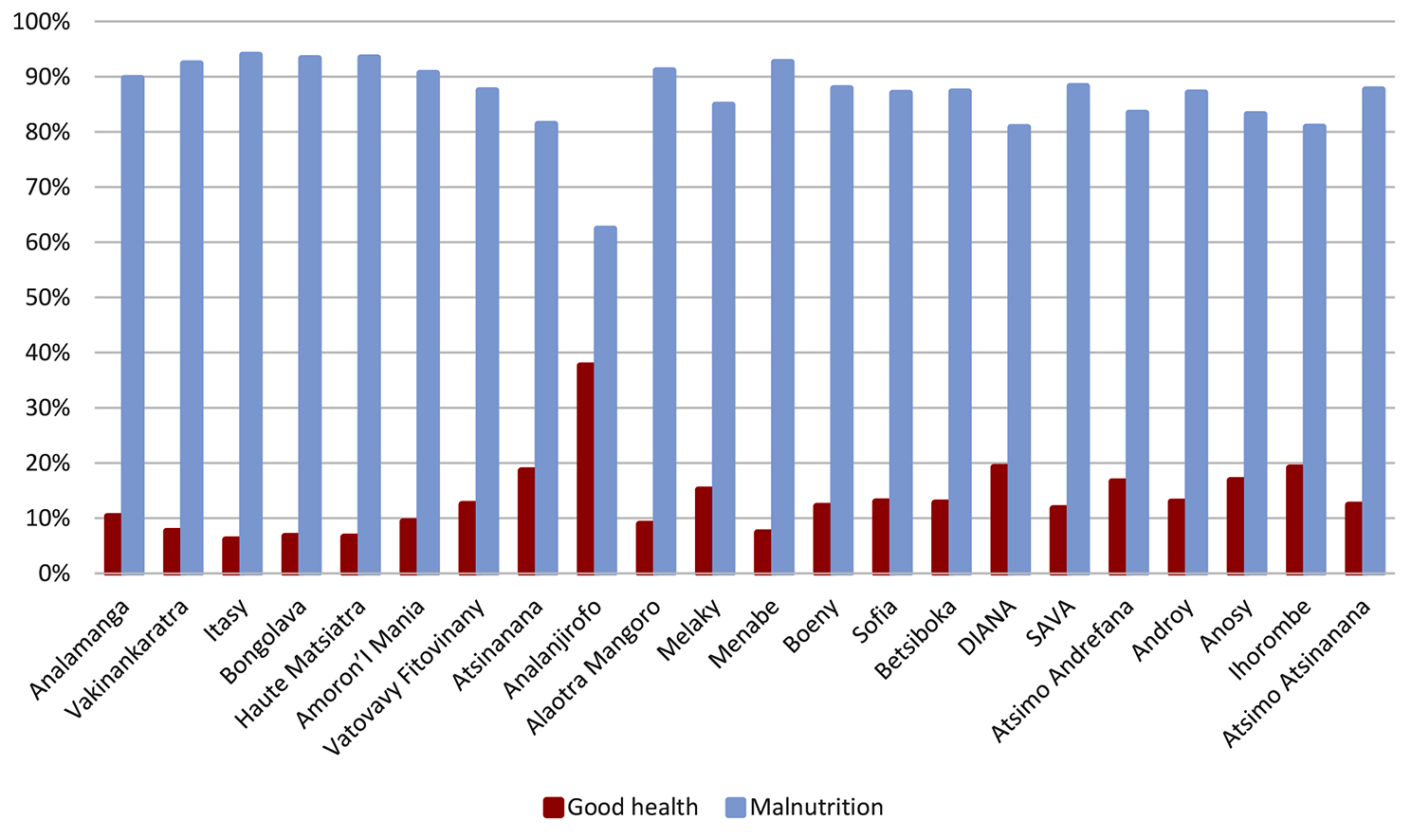
Prevalence of malnutrition by region in 2018 among children under five years. Sources: MICS, 2018; authors, 2024

Malnutrition rates differ significantly from one region to another (Fig. 1). The highest rates are found in the Itasy, Haute Matsiatra and Bongolava regions. These regions record 93.92%, 93.42% and 93.29% malnutrition, respectively. On the other hand, among all regions, the Analanjirofo region has the highest rate of good health (37.6%).

**Fig. 2:**
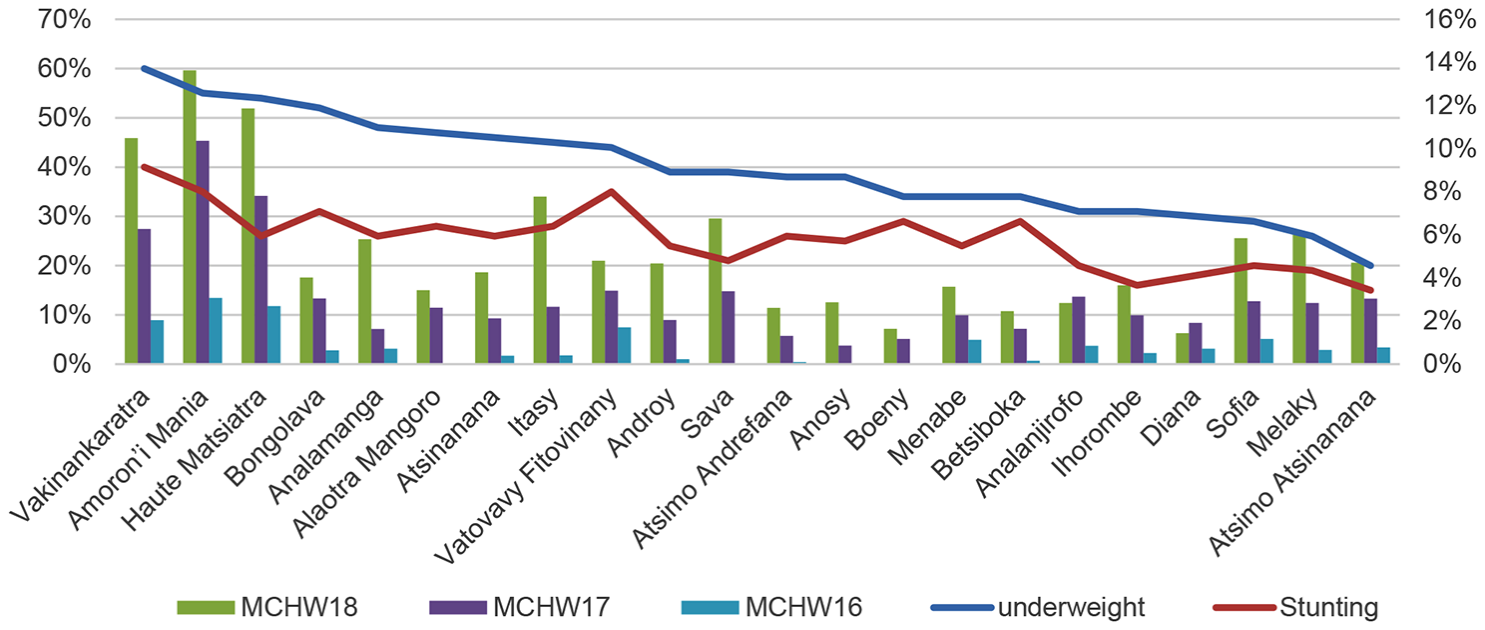
Underweight, stunting and MCHW by region in Madagascar. Sources: MICS, 2018; authors, 2024

The MICS database records participants in the 2016, 2017 and 2018 MCHW. In 2016, the Amoron’i Mania region had the highest participation rate (3.07%). In contrast, the Atsimo region recorded the lowest rate (0.10%). In addition, three regions did not organize MCHW in the same year: the Alaotra Mangoro, Boeny, and Sava regions. In 2017 and 2018, all regions organized MCHW. We note that the Amoron’i Mania and Haute Matsiatra regions are very active and have the highest rates during the 3 years of MCHW (10.36% in 2017 and 13.63% in 2018 for the Amoron’i Mania region; 7.8% in 2017 and 11.8% in 2018 for the Haute Matsiatra region). However, in terms of nutrition, these two regions are the most affected by malnutrition after the Vakinankaratra region, with a rate of 55% for the Amoron’ i Mania region and 54% for the Haute Matsiatra region (underweight) (Fig. 2).

Concerning mothers’ characteristics, 26.7% of the mothers were illiterate, and 48.6% had a primary level. However, 24.7% reached the secondary school level and above. The majority of mothers (89.1%) were in a couple.

Regarding the characteristics of the households surveyed, 29.4% live in extreme poverty and 24% in poverty. The lifestyles of most households remain precarious. Thus, the majority, 66.5%, live with an unimproved water source, 88.8% also live with an unimproved type of toilet, and 79.4% still use the traditional type of cooking fuel.

#### Results of the econometric models

The variables of interest are the use of MCHW. For Model 1, representing malnutrition, participating in MCHW 2016 decreases the probability of being malnourished. However, the effects are not immediately visible; the negative relationship is only significant for the year 2016. Very few children still participate in these public health activities (between 0.1 and 14% depending on the region). Then, an increase in participation compared to 2016 was noticed in 2017 and 2018. Thus, the significant values for 2017 in models 2, 3 and 4 can be explained by the fact that aware of the nutritional status of their children, parents decided to participate in the MCHW (Table 1).

**Table 1:** Effects of MCHW on the nutritional status of children under the age of 5 years in Madagascar

		Model 1	Model 2	Model 3	Model 4
		Malnutrition	HAZ	WAZ	WHZ
Variables		Odds ratio			
MCHW 2018		1.24 (0.19)	1.08 (0.13)	1.00 (0.90)	1.04 (0.52)
MCHW 2017		1.17 (0.47)	1.09 (0.29)	1.45*** (0.00)	1.74*** (0.00)
MCHW 2016		0.69** (0.05)	0.85** (0.03)	0.70*** (0.00)	0.57*** (0.00)
Child’s characteristics					
Sex		0.77*** (0.00)	0.86*** (0.00)	0.86*** (0.00)	0.84*** (0.00)
Age		1.08*** (0.00)	1.17*** (0.00)	1.21*** (0.00)	1.25*** (0.00)
Mother’s characteristics					
Education (ref = illiterate or preschool					
Primary		1.15 (0.12)	1.12*** (0.00)	1.09** (0.03)	0.95 (0.28)
Secondary and above		1.08 (0.50)	0.93 (0.21)	0.88** (0.02)	0.84*** (0.00)
Marital status		0.89 (0.33)	0.94 (0.24)	0.87*** (0.00)	0.86*** (0.01)
Household characteristics					
Urban/rural (ref = urban)		0.85 (0.13)	0.98 (0.71)	0.94 (0.21)	0.98 (0.68)
Income (ref = poorest)					
Poor		1.16 (0.15)	0.98 (0.62)	0.96 (0.38)	0.92 (0.10)
Average		1.23* (0.08)	0.99 (0.93)	0.86*** (0.00)	0.89* (0.06)
Rich		1.15 (0.31)	0.88** (0.05)	0.80*** (0.00)	0.93 (0.28)
Richest		1.38 (0.12)	0.76*** (0.00)	0.67*** (0.00)	0.76*** (0.00)
Source of drinking water		0.97 (0.76)	1.02 (0.67)	1.06 (0.13)	1.02 (0.61)
Toilets		0.78 (0.10)	0.90 (0.13)	0.94 (0.35)	0.90 (0.15)
Type of cooking		0.66*** (0.00)	0.95 (0.39)	0.94 (0.26)	1.00 (0.95)
Area (ref = Analamanga)					
Highlands	Vakinankaratra	0.97 (0.91)	1.15 (0.13)	1.23** (0.03)	0,99 (0,98)
	Itasy	2.41** (0.01)	0.90 (0.32)	0.93 (0.52)	1,09 (0,49)
	Bongolava	1.97** (0.02)	0.90 (0.32)	0.98 (0.87)	0,91 (0,42)
	Haute Matsiatra	1.31 (0.27)	0.98 (0.83)	0.94 (0.57)	0,88 (0,25)
	Amoron’i Mania	1.24 (0.43)	1.00 (0.94)	1.10 (0.38)	0,93 (0,54)
East	Vatovavy Fitovinany	1.03 (0.87)	0.71*** (0.00)	0.99 (0.95)	1,31*** (0,01)
	Atsinanana	1.32 (0.29)	0.82* (0.06)	0.79** (0.03)	0,87 (0,26)
	Analanjirofo	0.90 (0.70)	0.55*** (0.00)	0.67*** (0.00)	0,77** (0,04)
	Alaotra Mangoro	1.47 (0.17)	0.84 (0.10)	0.90 (0.35)	0,86 (0,21)
West	Melaky	0.98 (0.93)	0.33*** (0.00)	0.54*** (0.00)	0,85 (0,24)
	Menabe	1.17 (0.54)	0.44*** (0.00)	0.69*** (0.00)	1,22 (0,11)
North	Boeny	0.84 (0.51)	0.53*** (0.00)	0.77** (0.03)	0,93 (0,58)
	Sofia	0.83 (0.46)	0.50*** (0.00)	0.76** (0.02)	0,98 (0,92)
	Betsiboka	1.32 (0.28)	0.50*** (0.00)	0.83* (0.08)	1,33** (0,02)
	DIANA	0.56*** (0.00)	0.42*** (0.00)	0.61*** (0.00)	0,86 (0,24)
	SAVA	0.85 (0.57)	0.67*** (0.00)	0.81 (0.10)	0,88 (0,34)
	Atsimo Andrefana	1.63** (0.04)	0.64*** (0.00)	0.79** (0.02)	1,10 (0,35)
South	Androy	0.83 (0.43)	0.49*** (0.00)	0.66*** (0.00)	0,95 (0,68)
	Anôsy	1.20 (0.45)	0.53*** (0.00)	0.71*** (0.00)	1,11 (0,35)
	Ihorombe	0.74 (0.21)	0.41*** (0.00)	0.47*** (0.00)	0,75** (0,02)
	Atsimo Atsinanana	0.24*** (0.00)	0.17*** (0.00)	0.22*** (0.00)	0,42*** (0,00)
Constant		19.06			
Chi2		349.07***	1 079.13***	1 038.09***	874.59***
p-value Chi-2		0.00	0.00	0.00	0.00
Pseudo R2		0.06	0.03	0.03	0.04
Observations		14 431	14 431	14 431	14 431

*Note* The values in brackets are the p-values: ***, **,*, respectively significant at 1%, 5% and 10%. Sources: MICS, 2018; authors, 2024

Among the control variables, we found that malnutrition depends on the age and sex of the child. Malnutrition increases with age, and the probability of being malnourished is higher in boys than in girls. In addition, the search for nutritional supplements for parents would no longer be obvious, especially for poor households. Indeed, it was observed that children from households with a much higher standard of living are more likely not to suffer from stunting, underweight and wasting than the poorest. Then, the mother’s education and income explain stunting, underweight and wasting. The source of water and the type of toilet used do not affect malnutrition or its components. The use of modern fuels for cooking, on the other hand, decreases the probability of being malnourished. Finally, compared to the Analamanga region, the other regions of the Central Highlands (Itasy and Bongolava) and the Atsimo Andrefana region are much more affected by malnutrition. Moreover, it would seem that the probability of being affected by malnutrition, HAZ and WAZ is low in the Eastern, Western, Northern and Southern regions of Madagascar compared to the Central Highlands, where the rate of malnutrition is the highest. For WHZ, wasting, the values are much more scattered by region (Table 1).

The same results were obtained from the probit method (Appendix 1).

### Effects of rice price stabilisation policy on malnutrition

Following the analysis of MICS data, the nutritional status of children under the age of five years in Madagascar differs significantly from one region to another. The best nutritional status is observed in the Atsimo Atsinanana region, and the worst is observed in the highlands regions. However, in the Atsimo Atsinanana region, participation in MCHW appears to be low at 4% in 2018, 3.1% in 2017 and 0.6% in 2016. Moreover, when studying the characteristics of the Atsimo Atsinanana region, none of the variables considered in our study indicate particular specificities. One hypothesis is that it may be the rice price stabilisation policy that has therefore improved the nutritional situation in this area. Indeed, a stabilisation policy was implemented in 2017 and 2018. According to FAO (2018) data, an increase in rice consumption was observed in this region in 2018. This could partly explain the low malnutrition rate in this region.

In studying the relationship between stabilisation policy and malnutrition (Table 2), following the result of the Hausman test, a fixed effect model is selected. We found that price does not affect the malnutrition rate. The price decrease does not seem to apply to all regions, although it is a national policy. There are regions where the price is well below the average, such as Vakinankaratra, Itasy, and Haute Matsiatra. However, the rate of malnutrition in these regions remains above 50%. On the other hand, despite the policy, the price in the Analanjirofo and Melaky regions remains high; the rate of malnutrition in these regions is, however, less than 30%.

The same results are obtained when considering production multiplied by price. To the extent that it could be assumed that the policy via imports could have adverse effects on production, it should be noted that this is not the case, as the relationship between the quantity of rice produced and the quantity imported is not significant (*P* = 0.25). Therefore, agricultural policy alone has no effect on malnutrition.

Furthermore, our results show that the rate of malnutrition depends significantly on the rate of urbanization.

**Table 2:** Effect of price stabilisation policy on malnutrition within 22 regions in Madagascar from 2016 to 2019

Variables	Coefficient Model 1	Coefficient Model 2
Production	-0.02 (0.47)	-
Price	0.00 (0.11)	-
Production*Price	-	-0.01 (0.41)
HDI	-116.27 (0.10)	-100.92 (0.17)
Proportion urban rural	-0.58** (0.02)	-0.46* (0.07)
Constant	108.71*** (0.00)	101.21*** (0.00)
R^2^	0.61	0.56
Hausman test	36.34*** (0.00)	18.45*** (0.00)
Observations	88	88

*Note* The values in brackets are the p-value: ***, **, *, respectively significant at 1%, 5% and 10%. Sources: Global Lab Data; authors, 2024

## Discussion

This paper examines the effects of policies on nutrition outcomes. We consider the policies of immunization, vitamin A supplementation and deworming through MCHW and the rice price stabilisation policy.

Regarding the effect of nutrition policy, participating in MCHW 2016 decreases the probability of being malnourished. According to the WHO (2023), this is because micronutrients allow the body to produce enzymes and other substances essential for proper growth and development. Our results are in line with those of Semba et al. (2010) and Sudarsanam & Tharyan (2013) [15, 18]. Vitamin A is essential for vision and for the synthesis and metabolism of mucous membranes, bones, teeth and skin [15]. In addition, intestinal worms can cause illness, compromise children’s nutrition, cause micronutrient deficiencies and lead to anemia. Therefore, deworming helps to avoid these situations, and dewormed children also absorb vitamin A better [18]. Finally, vaccination protects against diseases. As these three activities are carried out during MCHW, it has been observed that they improve the nutritional status of children. However, as the relationship between the policy implemented in 2018 and malnutrition is not significant, it can be asserted that there is a certain lag in effects. The policy does not immediately influence the nutritional situation.

Regarding the other determinants, several studies have found the same results: malnutrition depends on the age and sex of the child [25, 26, 39]. This may be explained by the fact that morbidity and mortality are consistently higher in males than in females in early life, suggesting that boys are generally more vulnerable [40]. Concerning age, at the beginning of life, from 0 to 4 months, the child’s nutrition is ensured by breast milk. Then, from the weaning period, children become more mobile, which exposes them to contamination [39]. Then, it was observed that for HAZ and WAZ, mothers with primary education are more likely to have a malnourished child. Indeed, according to Reed et al. (1996), if resources remain insufficient, the level of education would not have the desired effect on malnutrition [41]. On the other hand, having a secondary education or higher reduces the probability of having an underweight and wasted child. In fact, the data showed that mothers with higher education levels are notably found in the richest category (26%) and the wealthiest category (36%). Mothers who are illiterate and with primary education are in the poorest categories. Finally, households with a high standard of living use modern fuels (24% of the rich and 55% of the richest), and using traditional fuels can cause infections in households, such as acute respiratory infections [42]. This fact can affect the nutritional state of children.

In studying the effects of the rice price stabilisation policy, rice being the staple food of Malagasy, a lack of relationship between this policy and the nutritional situation is found. However, according to Huang et al. (2016) and Parengam et al. (2010), rice, being rich in nutrients, could contribute to the fight against malnutrition, particularly in developing countries [10, 43]. However, although per capita rice consumption in Madagascar is high compared to other countries (130 kg per year versus 55 kg per year in the world according to the data of FAO, 2018), according to Andriamparany et al. (2021), diets are dominated by rice, so the consumption of fat-rich food groups and other products is low. Indeed, food diversification is not yet a priority for Malagasy households [44]. According to a study by USAID (2016), other foods, such as vegetables and livestock products, are intended for sale to obtain more rice [45]. According to this USAID (2016) study, only 10% of households reported consuming meat, eggs or dairy products. This situation thus explains the lack of effects of the rice price stabilisation policy on nutrition outcomes.

### Recommendations

Rice price stabilisation policy is not sufficient to ensure good nutrition for the population due to the lack of food diversification. Hence, there is a need for a direct nutrition policy that consists of providing the population with the additional micronutrients it needs, such as vitamin A, or a policy aimed at preventing diseases, such as vaccination. However, for these policies to be effective, awareness is needed to motivate the population to use them. Indeed, it was observed that the participation rate in MCHW is still low in Madagascar.

### Limitations

Our research has limitations in that the MICS does not include data on agricultural production. Therefore, two different databases are used for the two models relating to nutritional policy and the rice price stabilisation policy. For the price stabilisation models, four years are considered, as only information from 2016 to 2019 is available. Finally, variables may be omitted insofar as variables available in MICS and Global lab Data have been introduced into the models. However, this situation should not influence the quality of the models, as the same results were obtained in the different robustness tests.

## Conclusion

Malnutrition remains high in Madagascar despite the existence of various nutritional and agricultural policies. It was observed that nutritional policy, which is the MCHW, can significantly reduce the malnutrition rate. However, the effects are not immediate; the negative relationship is significant only for 2016, and participation in 2017 and 2018 had no effect. In addition, very few children still participate in these public health activities (between 0.1 and 14% depending on the region).

Furthermore, the rate of malnutrition varies significantly from one region to another. Given the low participation rate in MCHW, we wondered which policy would be effective, direct nutrition policy or an agricultural policy. Therefore, it is necessary to combine direct nutritional policies with agricultural policies to affect malnutrition. Thus, raising the awareness of the population to participate in the MCHW is necessary given the low participation rate. On the other hand, on the side of the agricultural policy, nutritional education and sensitization of the population to diversify its consumption is necessary.

### Electronic supplementary material

Below is the link to the electronic supplementary material.

Supplementary Material 1

## Data Availability

Data came from the database of the National Statistics Institute (2021). Then, we use Stata, so, the do.file is available.

## Footnotes

1 https://www.unicef.org/esa/reduce-stunting, consulted on 2nd February 2024.

2 Republic of Madagascar-Ministry of Public Health, “Evaluation of Vitamin A supplementation and deworming campaigns (2002 to 2006) and Mother and Child Health Weeks (2006 to 2011) in Madagascar”, Final Report July 2011.

## Abbreviations

HAZ: Height-for-age
MCHW: Mother and Child Health Week
MICS: Multiple Indicator Cluster Survey
NNP: National Nutrition Policy
SDGs: Sustainable Development Goals
WAZ: Weight-for-age
WHZ: Weight-for-height
