# Supplementary material for: Nutrition policy or price stabilisation policy: which policy is more effective for nutrition outcomes?
Source: BMC Nutr. 2024 May 11;10:72. doi: 10.1186/s40795-024-00882-6 (PMC11088095; doi:10.1186/s40795-024-00882-6)
Supplement: Supplementary file 1 — Supplementary Material 1 [file 40795_2024_882_MOESM1_ESM.docx]

**APPENDIX 1**

**Robustness tests:** Probit method.

|  | | Model 1 | Model 2 | Model 3 | Model 4 |
| --- | --- | --- | --- | --- | --- |
|  | | Malnutrition | HAZ | WAZ | WHZ |
| Variables | |  | | | |
| MCHW 2018 | | 0.12 (0.14) | 0.06 (0.28) | 0.04 (0.35) | 0.04 (0.35) |
| MCHW 2017 | | 0.11 (0.31) | 0.07 (0.31) | 0.27*** (0.00) | 0.42 (0.00) |
| MCHW 2016 | | -0.22** (0.01) | -0.14** (0.02) | -0.29*** (0.00) | -0.43 (0.00) |
| Child's characteristics | | | | | |
| Sex | | -0.12*** (0.00) | -0.14*** (0.00) | -0.17*** (0.00) | -0.09 (0.00) |
| Age | | 0.04***(0.00) | -0.14*** (0.00) | 0.16*** (0.00) | 0.17 (0.00) |
| Mother's characteristics | | | | | |
| Education (ref=illiterate or preschool | |  |  |  |  |
| Primary | | 0.06 (0.14) | 0.10*** (0.00) | 0.03 (0.36) | -0.02 (0.45) |
| Secondary and above | | 0.03 (0.56) | 0.09 (0.04) | -0.05 (0.23) | -0.09** (0.01) |
| Marital status | | -0.05 (0.38) | -0.03 (0.52) | -0.06 (0.16) | -0.07* (0.06) |
| Household characteristics | | | | | |
| Urban/rural (ref=urban) | | -0.07 (0.14) | -0.06 (0.15) | -0.08** (0.03) | -0.00 (0.88) |
| Income (ref=poorest) | |  |  |  |  |
| Poor | | 0.06 (0.22) | 0.05 (0.21) | -0.00 (0.93) | -0.03 (0.35) |
| Average | | 0.10* (0.07) | 0.05 (0.25) | -0.04 (0.35) | -0.06 (0.14) |
| Rich | | 0.07 (0.28) | 0.11** (0.04) | -0.06 (0.28) | -0.04 (0.35) |
| Richest | | 0.16 (0.10) | 0.06 (0.45) | -0.16** (0.03) | -0.14** (0.03) |
| Source of drinking water | | -0.02 (0.70) | 0.00 (0.90) | 0.06 (0.13) | 0.04 (0.16) |
| Toilets | | -0.12* (0.09) | -0.09* (0.08) | 0.00 (0.92) | -0.07 (0.10) |
| Type of cooking | | -0.21*** (0.00) | -0.16*** (0.00) | -0.14*** (0.00) | -0.02 (0.65) |
| Area (ref=Analamanga) | |  |  |  |  |
| Highlands | Vakinankaratra | -0.02 (0.88) | 0.09 (0.30) | 0.06 (0.44) | 0.05 (0.48) |
|  | Itasy | 0.38** (0.01) | 0.19* (0.07) | 0.24** (0.03) | 0.11 (0.18) |
|  | Bongolava | 0.30** (0.02) | 0.15 (0.14) | 0.14 (0.15) | -0.06 (0.43) |
|  | Haute Matsiatra | 0.12 (0.27) | 0.18 (0.06) | 0.17*(0.06) | -0.02 (0.74) |
|  | Amoron’i Mania | 0.09 (0.48) | -0.06 (0.55) | 0.07 (0.45) | -0.01 (0.87) |
| East | Vatovavy Fitovinany | 0.02 (0.85) | -0.16* (0.05) | 0.00 (0.95) | 0.04 (0.55) |
|  | Atsinanana | -0.14 (0.19) | -0.42*** (0.00) | -0.24*** (0.00) | -0.17** (0.03) |
|  | Analanjirofo | -0.74*** (0.00) | -1.00*** (0.00) | -0.87*** (0.00) | -0.50*** (0.00) |
|  | Alaotra Mangoro | 0.12 (0.30) | 0.04 (0.68) | -0.00 (0.97) | -0.00 (0.96) |
| West | Melaky | -0.06 (0.66) | -0.27*** (0.00) | -0.17* (0.08) | -0.15* (0.06) |
|  | Menabe | 0.17 (0.18) | 0.10 (0.31) | 0.05 (0.62) | -0.06 (0.42) |
| North | Boeny | -0.07 (0.54) | -0.13 (0.17) | -0.14 (0.14) | -0.06 (0.45) |
|  | Sofia | -0.09 (0.42) | -0.20** (0.03) | -0.06 (0.51) | 0.02 (0.76) |
|  | Betsiboka | 0.13 (0.27) | -0.17** (0.05) | -0.04 (0.61) | 0.12 (0.12) |
|  | DIANA | -0.02 (0.87) | -0.46*** (0.00) | -0.19* (0.06) | -0.09 (0.28) |
|  | SAVA | 0.22** (0.04) | -0.09 (0.28) | 0.10 (0.25) | 0.17** (0.02) |
|  | Atsimo Andrefana | -0.08 (0.41) | -0.36*** (0.00) | -0.15* (0.07) | 0.00 (0.97) |
| South | Androy | 0.08 (0.45) | -0.17* (0.06) | -0.03 (0.73) | 0.11 (0.13) |
|  | Anosy | 0.06 (0.61) | -0.36*** (0.00) | 0.01 (0.91) | 0.04 (0.65) |
|  | Ihorombe | -0.29** (0.01) | -0.42***(0.00) | -0.27*** (0.00) | -0.09 (0.28) |
|  | Atsimo Atsinanana | -0.08 (0.54) | -0.20* (0.06) | -0.09 (0.39) | -0.04 (0.62) |
| Constant | | 1.64*** (0.00) | 1.30 ***(0.00) | 1.13*** (0.00) | 0.39*** (0.00) |
| Chi2 | | 348.82*** (0.00) | 736.55*** (0.00) | 849.22*** (0.00) | 963.39*** (0.00) |
| Pseudo R2 | | 0.05 | 0.06 | 0.08 | 0.06 |
| Observations | | 14,431 | 14,431 | 14,431 | 14,431 |

*Note: the values in brackets are the p-values : ***, **,* respectively significant at 1%, 5% and 10%.*

Sources: MICS, 2018; authors, 2023
